# Out-of-hospital cardiac arrest research progress and challenges in Lithuania

**DOI:** 10.1016/j.resplu.2024.100664

**Published:** 2024-05-30

**Authors:** Deimante Baksevice, Linas Darginavicius, Gaile Damuleviciute, Monika Kunigonyte, Asta Krikscionaitiene, Egle Vaitkaitiene

**Affiliations:** a Department of Emergency Medicine, Lithuanian University of Health Sciences, A. Mickevičiaus g. 9, LT-44307 Kaunas, Lithuania; b Department of Disaster Medicine, Lithuanian University of Health Sciences, A. Mickevičiaus g. 9, LT-44307 Kaunas, Lithuania; c Department of Public Health, Lithuanian University of Health Sciences, A. Mickevičiaus g. 9, LT-44307 Kaunas, Lithuania

**Keywords:** Cardiac arrest, Cardiopulmonary resuscitation, Out-of-hospital cardiac arrest, Research, Research ethics, Methodology, OHCA, OHCA trial protocol, Utstein

## Abstract

**Aim:**

To present the evolution of data collection and analysis methods of out-of-hospital cardiac arrest (OHCA) research in Kaunas city, Lithuania, and discuss the challenges encountered.

**Methods:**

In late 2016, data collection began with a focus on 2016 data, following the Utstein 2014 template. The Kaunas city emergency medical services (EMS) station, which has a protocol dispatch system, pioneered the use of electronic submissions for the national EMS data collection form, making the research process more efficient. Most OHCA patients were treated in a tertiary university hospital which transitioned to electronic health record system in 2017, improving data accessibility. Throughout data collection significant efforts have been directed towards enhancing process efficiency and simplifying operations. As a result, the expansion of the Excel data table led to the creation of the ‘’resuscitation registry form’ ‘in 2018, which became operational in 2020.

**Results:**

The collected data were used in several observational studies to identify and better outcomes.

**Conclusion:**

Engaging in research on OHCA is difficult and poses many unique challenges owning to the urgency of the condition, complexity of legal and ethical considerations, and implications of any research intervention. The lack of a connection between the EMS and hospital electronic health record systems poses challenges for data collection. Legal and ethical complexities, including mandatory initiation of resuscitation and challenges in obtaining ethical approval, highlight the need for a comprehensive framework. This study aims transition the accumulated expertise into a nationally recognised registry for OHCA.

## Introduction

Out-of-hospital cardiac arrest (OHCA) is the sudden cessation of mechanical cardiac function that occurs outside of a medical facility^1^. In Kaunas, the incidence of emergency medical services (EMS)-treated OHCA is reported at 95.8 cases per 100,000 inhabitants per year.^2^ However, owning to limitations in the death coding system, comprehensive data on OHCA incidence rates across Lithuania are unavailable. OHCA is associated with a low survival rate and remains a leading cause of mortality, with nearly8% of the patients surviving.^2^, ^3^ Several variables affect the outcomes of OHCA, including the presence of witnesses, bystander-initiated cardiopulmonary resuscitation (CPR), initial heart rhythm, quality of hospital care, geographical location, and timing of OHCA events.^4^

EMS is critical in providing coordinated efforts in the chain of survival^5^ and bridging the crucial gap between the onset of cardiac arrest (CA) and advanced medical care.^6^ Moreover, EMS provides valuable data for OHCA research and quality improvement initiatives. Information collected on response times, interventions, and patient outcomes helps refine protocols and enhance overall emergency medical services.^7^

Research on OHCA presents methodological challenges that extend beyond the epidemiological domains. These include evaluating interventions, advancements in resuscitation techniques, and the integration of cutting-edge technologies. The topic of OHCA epidemiology and survival improvement is relevant and significant in Lithuania, as few systematic reports have been published in the last decade.^2^ There are almost no reports from other Baltic states, and none of them participated in the European registry of cardiac arrest (EuReCa) ONE, or EuReCa Two trials^3^, ^8^ also no data was submitted to International Liaison Committee on Resuscitation reports.^9^, ^10^ Prior to 2016, research on OHCA in Kaunas was scarce. This was partially owing to inefficiencies within the EMS, mainly caused by unsystematic manual data collection practices. Consequently, the reliability of the collected information is compromised, as it is frequently handwritten and prone to errors. However, significant systematic changes have occurred over the past decade, aligning with global research on OHCA. Through a comprehensive examination of methodological approaches, this study explores the intricacies of data collection methods and their advances and changes through the years, challenges, and limitations of conducting OHCA research in the city of Kaunas, Lithuania, which contributes to the advancement of OHCA knowledge and, ultimately, improves patient outcomes.

## Methods

### Setting

**Fig. 1:**
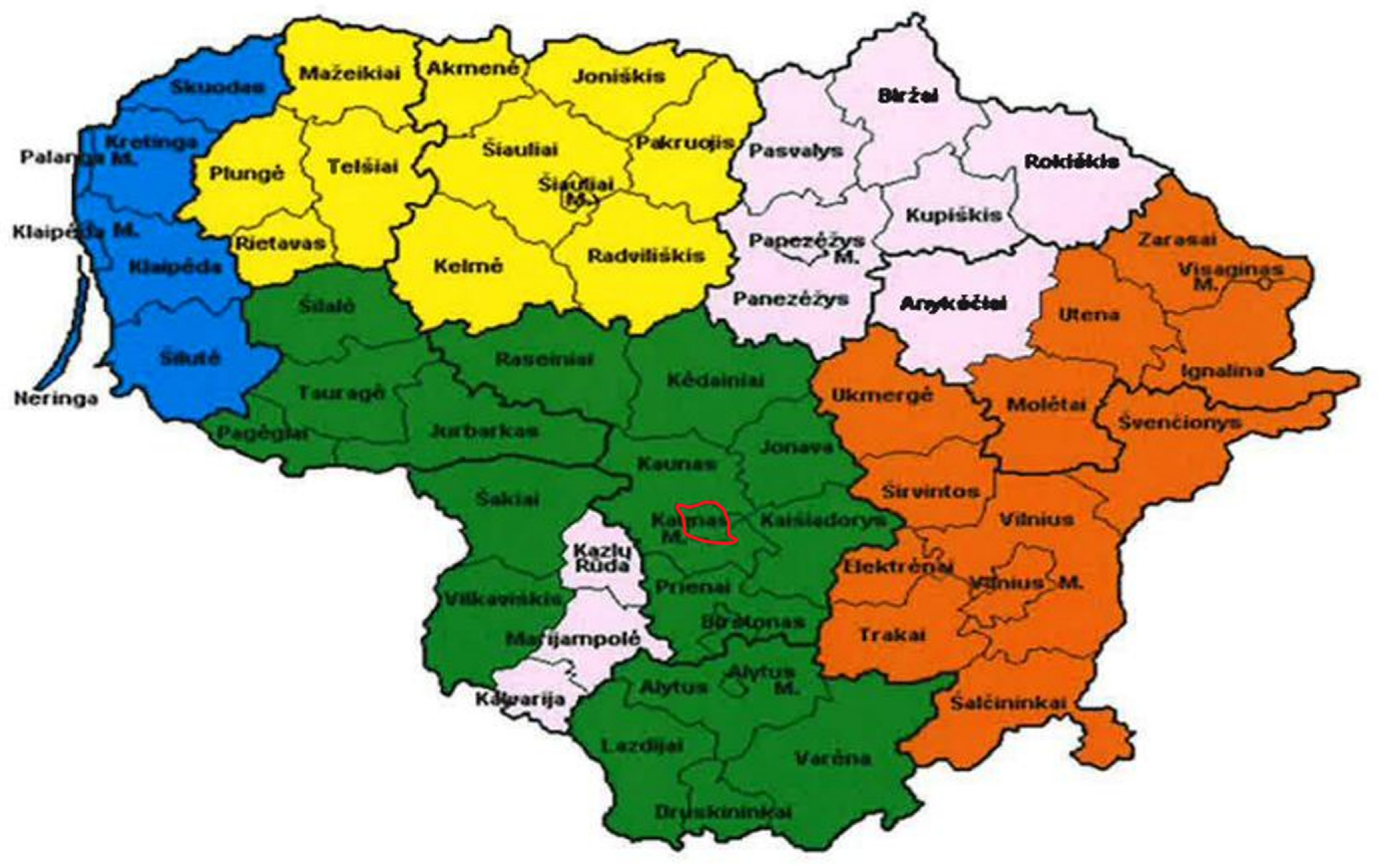
The Kaunas city emergency medical services station dispatch centre control area is marked in green; the area marked in red is Kaunas city. (For interpretation of the references to colour in this figure legend, the reader is referred to the web version of this article.)

Kaunas is the second- largest city in Lithuania after the capital city of Vilnius. It is located in southern Lithuania, with a population of 304,198 people^11^ and an area of 157 km^2^.^12^ The Kaunas city EMS, one of five dispatch centres, serves 900,000 people (see Fig. 1) in 2021, they received 237,717 calls and dispatched teams to assist 78,794 individuals.^13^

**Fig. 2:**
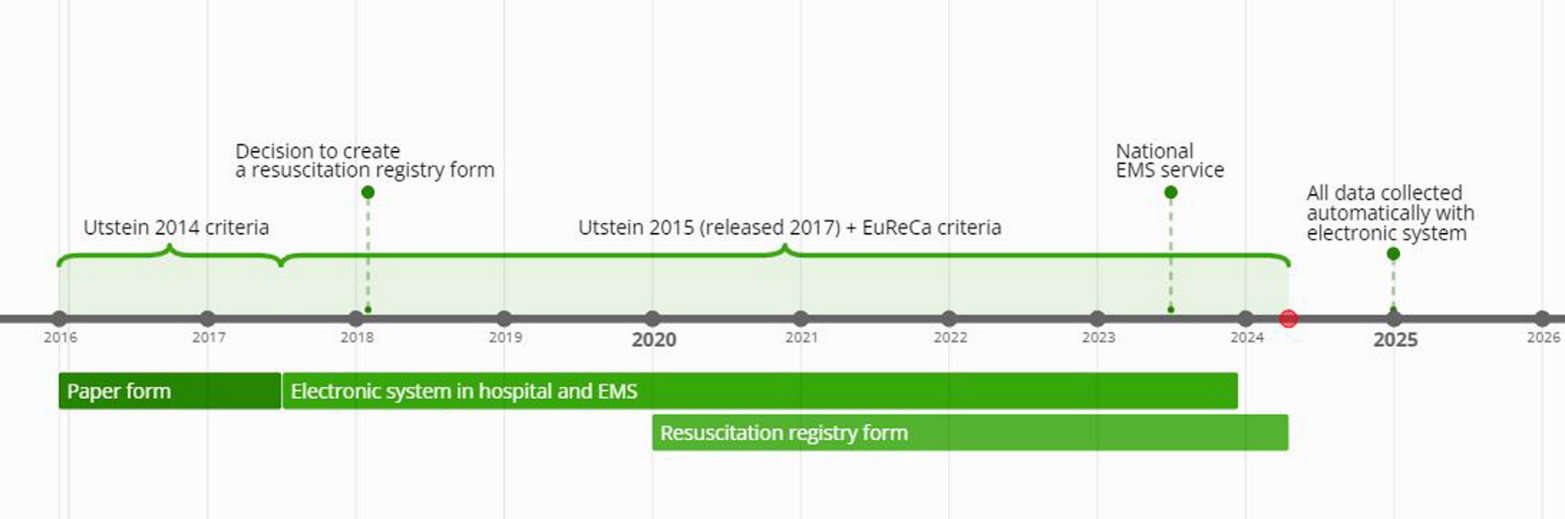
Evolution of emergency medical services documentation: a timeline.

In this section, we elucidate the well-established process of collecting and analyzing data, highlighting the significant changes that have occurred over time (see Fig. 2). Our data sources included the Kaunas city EMS database records, Kaunas city EMS quality manager's call records audit, and hospital electronic health records.

The Kaunas city EMS station operates a comprehensive protocol-based dispatch system and a unique two-unit principle. When a dispatcher suspects the need for resuscitation, as determined through a protocol encompassing enquiries, such as the patient's breathing status, pulse presence, and other relevant criteria, they promptly activate two EMS teams: the nearest unit capable of administering basic life support, which consists of paramedics or a nurse, and the paramedic and advanced life support unit, typically staffed with a physician and, competent to perform advanced life support.

### Ethical approval

Ethical approval for this research was issued on 13 July 2020 by the Lithuanian University of Health Sciences (No. BE-2-270). Consent for participation was not required. Also the research was registered in the clinicaltrials.gov database with the identifier NCT04784117 and the Unique Protocol ID LITOHCA From 2016 to 2021.

### Evolution of data collection

The initial stage of data collection occurred between late 2016 and early 2017, with a primary focus on data from 2016. We followed the Utstein 2014 template, and meticulously extracted and manually recorded data in Excel spreadsheets. Research data was only collected from the Kaunas city area and, not from all calls managed by the Kaunas city EMS station. Cases were identified based on the lack of a pulse by the first team at the scene. Although there have been solitary instances of return of spontaneous circulation (ROSC) achieved by bystander CPR and automated external defibrillator (AED) shock, these were registered and not included in analysis.

In 2014, Lithuania has adopted a nationally approved protocol-based EMS data collection form, enabling comprehensive documentation and the extraction of data on all resuscitation cases. A notable feature of the Kaunas city EMS station is its pioneering electronic submission of data to the national EMS data collection form, which significantly improved data collection efficiency, both clinically and for research.

In Kaunas city, despite the presence of four hospitals with emergency departments, the vast majority of OHCA patients (87%) visited tertiary university hospital with coronary angiography capabilities and about 41% underwent coronary angiography.^2^ Importantly, the university hospital transitioned from a paper-based health record system to an electronic one in the middle of 2017, signifying a substantial improvement in data accessibility which means that data were collected from all Kaunas city hospitals.

Following the initial year of data collection according to the 2014 Utstein template it became apparent that the dataset was insufficient for comparative analysis, particularly in context of the EuReCa ONE study,^8^) an inaugural multi-centre European OHCA study. Data is collected and examined on 27 core and supplemental variables regarding the system (population served, number of CAs attended, number of resuscitation attempted and not attempted, system description), the dispatcher (dispatcher identified presence of CA, dispatcher provided the CPR instructions), patient (age, sex, witnessed arrest, arrest location, bystander response, first monitored rhythm, aetiology), process (response times, defibrillation time, provision of targeted temperature management, drugs, performance of coronary angiography, number of occluded arteries), and outcomes (prehospital ROSC, survived event, survival to hospital discharge, 1-year survival, transport to hospital, neurological outcome at discharge and discharge location). To overcome this limitation, the Excel data table was expanded to align with the EuReCa criteria, and additional variables of interest were incorporated into the data collection protocol (see Table 1).

**Table 1:** Newly added and examined variables in the expansion of the Utstein template.

Variable	EuReCa	Lithuania
EMS witnessed cardiac arrest in a helicopter	+	
Not attempted bystander CPR	+	
Responder CPR by ‘‘firefighter’’or, ‘‘police officer’’		+
Number of responders ‘‘one, two or more, not recorded, not applicable’’		+
Gender of responder:‘‘both’’		+
Time and date of AED	+	
Medication used in resuscitation ‘‘other’’		+
Reason for OHCA ‘‘other intoxication’’, ‘‘other’’		+
EMS defibrillator: ‘‘yes, applied without defibrillation’’or, ‘‘yes, applied with defibrillation’’		+
Defibrillator used: ‘‘manual’’or, ‘‘AED’’		+
EMS airway management ‘‘other’’, ‘‘jaw thrust’’		+
EMS ventilation ‘‘bag valve mask’’, ‘‘mechanical ventilator’’or, ‘‘not applicable’’		+

The acquisition of this comprehensive dataset necessitated a significant amount of manual labour, leading to the creation of the ‘‘resuscitation registry form’’ in 2018, which was operationalized in the Kaunas city EMS station in 2020 (see Appendix). The form was designed to be completed by EMS providers at the scene of an OHCA, gathering essential information about the patient and the circumstances of the OHCA event, as well as the administered treatment and precise times of administration. The primary aim of this form is to streamline the quality assurance process for EMS service providers and facilitate data collection for researchers. Fields in the form that are the same as those in the main EMS documentation form are obligatory and automatically duplicated in both forms. All other fields were axillary and not obligatory; however, the filling percentage was monitored. Outcome data were collected manually from the hospital database and the 1-year survival rate was collected from the Lithuanian Health Information Centre of Institute of Hygiene, which is responsible for the national death statistics in Lithuania. Although the Cerebral Performance Category (CPC) scale does not closely correlate with discharge location,^14^ we presumed that discharge to a hospice indicates poor neurological outcomes, whereas discharge to home suggests better outcomes. Regrettably, the use of CPC or modified Rankin scales has not been adopted locally as part of standard care.

Notably, EMS services in Lithuania are provided by 49 EMS facilities, which consist of 19 public health care facilities under the authority of municipal councils, 28 units of public health care facilities, and 2 private companies. Before 1 July 2023, these services were autonomously administered across 49 stations, each with the freedom to choose among 5 dispatch centres and whether to employ paper-based or electronic EMS data collection methods, among other operational considerations. Following a governmental resolution, all 49 stations were consolidated into a single National EMS service, with the primary objective of standardizing EMS practices nationwide. Until now, we have collected data from only one of 49 facilities, with this change, there is potential to expand data collection to all OHCA cases in Lithuania.

In addition, the EMS quality manager conducted audits of dispatch-assisted CPR calls, providing valuable data, such as the time and details of events, bystander information, and actions taken for research purposes. The Kaunas city EMS station, in particular, utilised the standard MPDS ProQA® system, recognized for its fully protocol-based framework and a dedicated auditing tool that evaluated various aspects, such as the recognition of CA by the dispatcher, caller emotions, and more.

Throughout data collection, significant efforts have been directed towards enhancing process efficiency and simplifying operations. Notably, this ongoing evolution is progressively leading us towards the ultimate goal of fully automating the analysis process, with all data being collected electronically. However, it is important to acknowledge that a key challenge remains unresolved as the EMS and hospital data continue to exist as separate entities, necessitating manual integration.

## Results

From 2016 to 2021, data on a total of 1,588 cases were collected. The median age of the patients was 72 years, and approximately 67% were male. The estimated time for the EMS arrival to the scene was around 8 min in Kaunas city. ROSC on the scene in 2020–2021 was reached in 22,7% of cases.^7^ The initial rhythm was shockable in 27.6% of all cases and non-shockable in 68.5%.^2^ The data collected from the previously stated form is used in a various research endeavours on OHCA in Lithuania, including but not restricting to survival rates, both external and internal factors contributing to ROSC, and the importance of airway management in resuscitative efforts.

## Discussion

Researching OHCA is a challenging task that involves unique obstacles owning to the urgency of the condition and legal and ethical considerations of any research intervention. In this study, we discuss the four main challenges encountered.

First, it is important to note that the data collection form we used was only implemented at one of the 49 stations. This emphasizes the need to expand form utilization in other EMS stations to collect a more diverse and representative sample of data^15^). Although all stations have been consolidated into one National EMS service, which should improve the amount of data collected nationwide, it presents new challenges. The shift towards a centralized system can affect the diversity of contexts and practices within individual stations. In addition, there are different milestones in reaching patients in urban and rural territories, whereas all our previous studies were done in one urban territory. Therefore, it is crucial to implement a clear and standardised resuscitation registry form-filling process to ensure consistent and reliable data collection.^16^, ^17^ Standardising data collection can help us compare resuscitation results between local and international stations. Moreover, it can improve the EMS network as information is collected not only from patient and resuscitation data but also from the system itself, such as dispatch and arrival times.^18^

Second, a significant portion of data collection is currently performed manually, which involves a meticulous analysis of forms and audio recordings by a researcher. Data collected from audio recordings include dispatcher recognition of OHCA, dispatcher assisted CPR, time to instruction and chest compressions, bystander performance of CPR, time to CPR termination or handover. As mentioned previously, collecting the necessary information for research requires accessing at least three different databases and manually gathering information for each case. This approach may introduce bias towards certain sources and raise concerns regarding the accuracy of the process. Furthermore, this method is time consuming and inefficient.

Some studies highlight the advantage of individually analysing each audio recording; this approach may primarily serve as an effective method for verifying information.^19^ Several countries are now experimenting with artificial intelligence recognition technology that can alert emergency responders to OHCA incidents and even collect patient data to streamline data collection and improve accuracy.^20^

Third, we analysed data from all resuscitation instances, indicating that Lithuania lacks a ‘do not resuscitate’ law. EMS paramedics are required to initiate resuscitation measures in all cases due to the country's legal background. The pronouncement of death can only be made by nurses or doctors, who usually arrive later at the scene, as the closest EMS car is always dispatched to the scene. This framework ensures that life-saving interventions are administered promptly, because Lithuania places high value on preserving life at all costs.^21^ However, this structure and decision-making process raises concerns about patient autonomy, as it disregards the right to express and make decisions especially regarding end-of-life care in cases of terminal illness.^22^ Additionally, it increases the number of unsuccessful resuscitations and the incidence rate per 100,000 inhabitants/year, leading to prolonged suffering, increased medical interventions, and financial burden on the healthcare system, particularly for terminally ill patients in prehospital settings.^23^

Finally, obtaining ethical and legal approval for a study can be a time-consuming and challenging task because of patient information protection laws that require informed consent from patient or their relatives. In Lithuania, the Lithuanian Bioethics Committee is responsible for ensuring that all research is ethical and legal, and in most cases, approval is sufficient for any medical research. However, obtaining access to hospitals or EMS databases necessitates institutional approval, with decisions being influenced by national committees. This challenge is a well-recognized and frequently discussed one.^24^ Restricting data collection to patients who could provide informed consent may introduce bias and limit the diversity and representation of the study, which could potentially harm future patients. Furthermore, some patients may wish to contribute to research and benefit society even if they are unable to provide consent.^25^ Alternatives to informed consent, such as waived or deferred consent, have been used in many studies on sudden CA. One study from the Netherlands emphasised that seeking consent after an event may take several days or even months; in some cases, such as sudden CA, seeking consent may be impractical.^26^ Therefore, there is a critical need for an ethical and context-sensitive framework for collecting, sharing, and using data from patients in critical conditions, such as OHCA.^25^

## Conclusions

The OHCA research in Lithuania made significant strides in all areas, particularly data collection. The manual recording process has been replaced by an electronic ‘resuscitation registry form,’ streamlining EMS procedures. However, our research was limited to only one EMS station, which could hinder the generalisability of the results. The recent consolidation of the National EMS service presents opportunities for improved data collection, however standardization challenges may arise. Manual data collection has drawbacks, although technological progress may help to improve efficiency and accuracy. Moving towards full automation aligns with efficiency goals, nonetheless challenges must be addressed to ensure valid and applicable emergency care research. Legal and ethical complexities, such as the absence of a ‘do not resuscitate’ law and mandatory resuscitation initiation, require attention. A comprehensive framework is crucial to ensure ethical data use and to address the unique challenges in OHCA research.

## Funding

This research did not receive any specific grant from funding agencies in the public, commercial, or not-for-profit sectors.

## CRediT authorship contribution statement

**Deimante Baksevice:** Writing – review & editing, Writing – original draft, Investigation, Conceptualization. **Linas Darginavicius:** Writing – review & editing, Writing – original draft, Visualization, Supervision, Investigation, Conceptualization. **Gaile Damuleviciute:** Writing – review & editing, Writing – original draft. **Monika Kunigonyte:** Writing – review & editing, Writing – original draft. **Asta Krikscionaitiene:** Writing – review & editing, Supervision, Investigation, Conceptualization. **Egle Vaitkaitiene:** Supervision, Investigation, Conceptualization.

## Declaration of competing interest

The authors declare that they have no known competing financial interests or personal relationships that could have appeared to influence the work reported in this paper.
