# Supplementary material for: Out-of-hospital cardiac arrest research progress and challenges in Lithuania
Source: Resusc Plus. 2024 May 30;19:100664. doi: 10.1016/j.resplu.2024.100664 (PMC11170472; doi:10.1016/j.resplu.2024.100664)
Supplement: Supplementary Data 1 [file mmc1.pdf]

CARDIAC ARREST REGISTRY

PART A: DEMOGRAPHIC INFORMATION

1. Adress (Where Arrest Occured)

2. First Name

3. Last Name

4. Gender
- ☐ Male
- ☐ Female

5. Age
- ☐ Days
- ☐ Months
- ☐ Years

6. Date of Birth _ _ _ _ - _ _ - _ _

7. Medical History/Allergies
- ☐ Asthma
- ☐ Hypertension
- ☐ COPD
- ☐ Arrhythmia
- ☐ Renal
- ☐ Unknown
- ☐ TB
- ☐ Diabetes
- ☐ CHD (MI)
- ☐ Cancer
- ☐ Stroke

8. Dispatch Centre

9. EMS station

10. Incident # _ - _ - _

11. Patient #

12. Dispatch ID

13. EMS crew #

14. Date 2 0 _ _ - _ _ - _ _

15. Chief of Crew #

16. 2nd Member of Crew #

17. 3rd Member of Crew #

18. 4th Member of Crew #

19. Time call received at dispatch center _ _ : _ _

20. Time Ambulance dispatched _ _ : _ _

21. Time of Departure _ _ : _ _

22. Time of Arrival _ _ : _ _

23. Transportation Time _ _ : _ _

24. Time of Arrival to ED _ _ : _ _

25. Completion Time _ _ : _ _

26. Time of Return _ _ : _ _

PART B: RESUSCITATION BEFORE EMS

27. Location Type
- ☐ Home/Residence
- ☐ Healthcare Facility/Nursing Home
- ☐ Workplace
- ☐ Street
- ☐ Public Building
- ☐ Sports and Entertainment Area
- ☐ Learning Institution
- ☐ Whitnessed during HEMS tranfer
- ☐ Other

28. Arrest Whitnessed by
- ☐ Family Members
- ☐ Police
- ☐ FRS
- ☐ Medical Provider
- ☐ Dispatched First Aid Provider
- ☐ Other
- ☐ Unwitnessed Arrest

29. Presumed Cardiac Arrest Etiology
- ☐ Heart Disease
- ☐ Trauma
- ☐ Drowning
- ☐ Drug Poisoning
- ☐ Other poisoning
- ☐ Other diseases (exept heart diseases)
- ☐ Asphyxia (external cause)
- ☐ Electrocution

30. CPR before EMS
- ☐ Yes
- ☐ No

31. Who Initiated CPR
- ☐ Bystander
- ☐ Family Members
- ☐ Police
- ☐ FRS
- ☐ Medical Provider
- ☐ Dispatched First Aid Provider
- ☐ Other

32. Number of CPR providers
- ☐ One
- ☐ Two and more
- ☐ Unknown

33. Type of Bystander CPR Provided
- ☐ Compressions Only
- ☐ Compressions and Ventilations
- ☐ Ventilations Only

34. Were Dispatcher CPR instructions provided
- ☐ Yes
- ☐ No
- ☐ Unknown

35. Was an AED applied prior to EMS arrival
- ☐ Yes, with defibrillation
- ☐ Yes, without defibrillation (or not required)
- ☐ No

36. Who First Defibrillated the Patient
- ☐ Bystander
- ☐ Family Members
- ☐ Police
- ☐ FRS
- ☐ Medical Provider
- ☐ Dispatched First Aid Provider
- ☐ Other

37. Number of Defibrillations

\# _ _ ☐ Unknown

38. Age of Bystander CPR Provider

Age _ _ _ ☐ Male ☐ Female

Age _ _ _ ☐ Male ☐ Female

PART C: EMS RESUSCITATION

39. First Arrest Rhythm of Patient
- ☐ Ventricular Fibrillation
- ☐ Pulseless Ventricular Tachycardia
- ☐ PEA
- ☐ Asystole
- ☐ Bradycardia
- ☐ AED instructed to defibrillate
- ☐ AED did not instruct to defibrillate

40. ROSC present at the scene (detectable pulse >5 min)
- ☐ Yes
- ☐ No

41. End of Event
- ☐ Pulse on ED arrival
- ☐ Ongoing resuscitation in ED (Mechanical CPR)
- ☐ Pronounced Dead in the Field

42. When did ROSC first occur
- ☐ Before EMS
- ☐ After EMS Basic Life Support
- ☐ After EMS Advanced Life Support

43. Defibrillation used
- ☐ Yes
- ☐ No

44. Number of times defibrillated

45. Time of Heart Arrest _ _ : _ _

46. Time of Start of Resuscitation _ _ : _ _

47. Time of First Defibrillation _ _ : _ _

48. Time of End of Resuscitation _ _ : _ _

49. EMS Interventions

Time	Crew Member	Procedure ID	Medication ID	Administration ID	Admn. Intens. ID	Size	In total	Status	Comments
:									
:									
:									
:									
:									
:									
:									
:									
:									

50. Automatic CPR feedback device used:
- ☐ Yes
- ☐ No

51. STEMI
- ☐ Yes
- ☐ No
- ☐ ECG was not recorded

52. Transportation. Selection of Hospital
- ☐ Closest Hospital
- ☐ Patient/Family Members Choice
- ☐ Specialised Hospital

Destinaton Hospital

Department
